# A Mosaic Nanoparticle Vaccine Elicits Potent Mucosal Immune Response with Significant Cross‐Protection Activity against Multiple SARS‐CoV‐2 Sublineages

**DOI:** 10.1002/advs.202301034

**Published:** 2023-08-01

**Authors:** Xiantao Zhang, Shijian Wu, Jie Liu, Ran Chen, Yongli Zhang, Yingtong Lin, Zhihui Xi, Jieyi Deng, Zeyu Pu, Chaofeng Liang, Jinzhu Feng, Rong Li, Keming Lin, Mo Zhou, Yingying Liu, Xu Zhang, Bingfeng Liu, Yiwen Zhang, Xin He, Hui Zhang

**Affiliations:** ^1^ Institute of Human Virology Department of Pathogen Biology and Biosecurity Key Laboratory of Tropical Disease Control of Ministry of Education Zhongshan School of Medicine Sun Yat‐sen University Guangzhou 510080 China; ^2^ Guangzhou National Laboratory Bio‐Island Guangzhou 510320 China

**Keywords:** broad neutralizing antibody, mosaic nanoparticle vaccine, mucosal immune responses, SARS‐CoV‐2, universal vaccines

## Abstract

Because of the rapid mutation and high airborne transmission of SARS‐CoV‐2, a universal vaccine preventing the infection in the upper respiratory tract is particularly urgent. Here, a mosaic receptor‐binding domain (RBD) nanoparticle (NP) vaccine is developed, which induces more RBD‐targeted type IV neutralizing antibodies (NAbs) and exhibits broad cross‐protective activity against multiple SARS‐CoV‐2 sublineages including the newly‐emerged BF.7, BQ.1, XBB. As several T‐cell‐reactive epitopes, which are highly conserved in sarbecoviruses, are displayed on the NP surface, it also provokes potent and cross‐reactive cellular immune responses in the respiratory tissue. Through intranasal delivery, it elicits robust mucosal immune responses and full protection without any adjuvants. Therefore, this intranasal mosaic NP vaccine can be further developed as a pan‐sarbecovirus vaccine to block the viral entrance from the upper respiratory tract.

## Graphical abstract

A mosaic receptor‐binding domain‐based nanoparticle vaccine is generated to elicit broad neutralizing against SARS‐CoV‐2 virus. The cross‐reactive cellular immune responses are further robustly induced by combinational conjugation of conserved T‐cell epitopes from sarbecoviruses. Through intranasal delivery without any adjuvants, our vaccine also elicits potent mucosal immune responses to combat the virus infection.

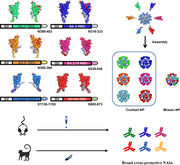

## 1 Introduction

COVID‐19 has become a global pandemic due to the SARS‐CoV‐2 infection.^[^ ^1^ ^]^ In addition to SARS‐CoV‐2, the 229E, OC43, NL63, and HKU1 lineages in the coronaviruses family cause mild upper respiratory infection, while SARS‐CoV and MERS‐CoV infections resulted in serious diseases.^[^ ^2^ ^]^ To date, there have been more than 100 SARS‐CoV‐2 vaccines in clinical prophylactics and trials.^[^ ^3^ ^]^ Although various vaccines based upon the early strain have played an important role in controlling the pandemic, SARS‐CoV‐2, especially its newest Omicron strain, exhibits the fastest transmission and high mutation rates and leads to significant immune evasion and vaccine invalidity.^[^ ^4^ ^]^ It is expected that pan‐CoV vaccines could be developed fto prevent newly‐emerged SARS‐CoV‐2 sublineages and other sarbecoviruses.^[^ ^5^ ^]^ Some variant‐proof and pan‐sarbecovirus vaccines are currently in the early clinical or close to early clinical stage and are likely to be available in the near future.^[^ ^3^ ^]^ Conversely, although it has been well known that SARS‐CoV‐2 replication takes place primarily in the upper respiratory tract, almost all the current vaccines cannot induce effective respiratory mucosal immunity and block the viral invasion from the forefront of infection.^[^ ^6^, ^7^ ^]^ It is necessary to develop a universal vaccine capable of preventing infection in the upper respiratory tract.

Two main research strategies have been engaged to develop the universal vaccine. One is to combine divergent antigens from multiple coronavirus subgroups together to stimulate immune protection against multiple coronaviruses, which is currently the most used strategy.^[^ ^8^ ^]^ Another is to look for conserved protein structures common in multiple coronaviruses and utilize them as antigens to develop vaccines.^[^ ^9^ ^]^ Previously we equipped the receptor binding domain (RBD) of Spike protein from SARS‐CoV‐2 on the surface of helicobacter pylori ferritin (HPF) scaffold to generate an RBD‐subunit‐based nanoparticle (NP) vaccine, which elicited a robust immune response in both mice and rhesus macaques.^[^ ^10^ ^]^ Several other groups have also developed similar NP vaccines.^[^ ^11^ ^]^ Further, mosaic RBD or spike NP vaccine, comprised of conjugating RBD of SARS‐CoV, SARS‐CoV‐2, and several sarbecoviruses, or spike proteins from the SARS‐CoV‐2 three variants.^[^ ^8^, ^12^ ^]^ In this study, we designed a mosaic RBD NP vaccine, which not only induced potent humoral and cellular immune responses but also elicited robust mucosal immunity with broad cross‐protective activity when inoculated intranasally without adding any adjuvants. This novel intranasal vaccine is capable of overcoming the immune evasion caused by severe mutations of the SARS‐CoV‐2 variants and blocking the viral invasion from the upper respiratory tract.

## 2 Results

### 2.1 Construction and the Immunization Effectiveness of the Mosaic RBD Nanoparticle Vaccines

**Figure 1:**
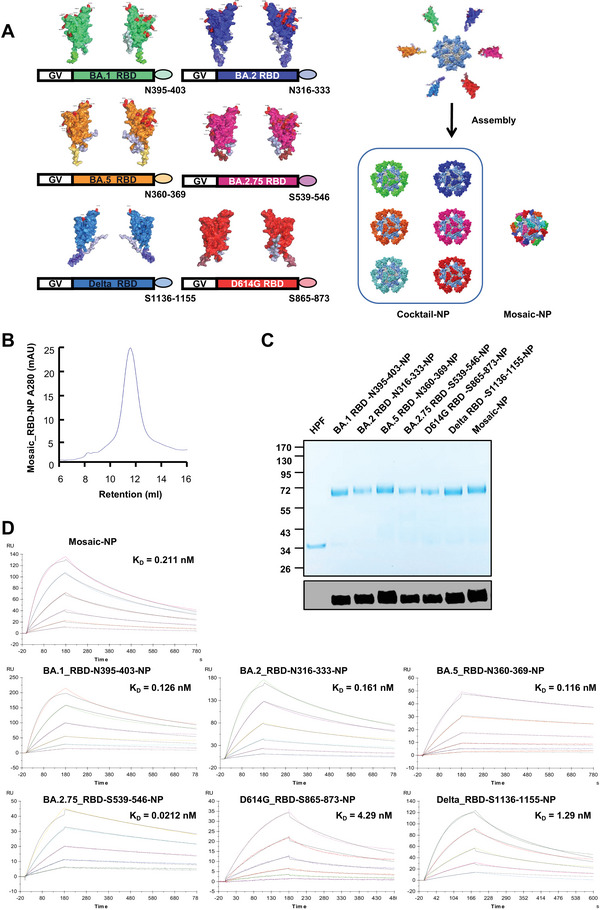
Characterization and purification of the mosaic RBD nanoparticle vaccines. A) A Schematic diagram of mosaic RBD nanoparticle vaccines. The RBD of BA.1, BA.2, BA.5, BA.2.75, Delta, and D614G variants were labeled with six highly conserved T cell epitope peptides from the Sarbecovirus N or S proteins, respectively. mosaic NP contained the six RBD mentioned above fused to the Gv tag and was assembled with Sd‐HPF in vitro. Cocktail NP includes the six individual RBD NPs from the SARS‐CoV‐2 prototype variants mentioned above. AlphaFold simulated the structure of RBD NP. The figure was created with PyMOL‐2.5.2 and UCSF ChimeraX‐1.16. B) The size exclusion chromatography of mosaic RBD‐NP. The UV absorptions at 280 were shown. The retention volume represented the peak of the nanoparticle. C) Coomassie blue staining(top)of BA.1 RBD‐N395‐403‐NP, BA.2 RBD‐N316‐333‐NP, BA.5 RBD‐N360‐369‐NP, BA.2.75 RBD‐S539‐546‐NP, D614G RBD‐S865‐873‐NP, Delta RBD‐S1136‐1155‐NP, and mosaic RBD‐NP. The expression and purity of each protein were confirmed by western blotting with RBD antibodies (bottom). D) The binding affinity of each RBD NP to hACE2 protein was analyzed with Surface plasmon resonance (SPR). The displayed K_D_ value shown was the average of three independent experiments.

We chose the RBDs of four significant sublineages in the Omicron family, including BA.1, BA.2, BA.5, and BA.2.75, as well as Delta and the early strain (D614G), as our primary antigens, and displayed these six RBDs on one NP surface (**Figure** **1**A) (Figure S1A, Supporting Information). To quantitate the amounts of various RBDs, we labeled the RBDs by fusing six highly conserved T cell epitope sequences from the sarbecovirus N or S proteins, respectively (Figure S1B, Supporting Information).^[^ ^9^ ^]^ Thus, these epitope sequences not only mark and quantitate various RBDs but also improves the cellular immune response and the broad protection against sarbecoviruses. The expression and purification of the corresponding nanoparticle conjugates were performed as shown in Figure 1B,C (Figure S2A,B, Supporting Information). The binding affinity and kinetics between RBD‐NPs and hACE2 did not change significantly, indicating that the epitopes on the NPs were correctly folded and exposed (Figure 1D). To compare the efficacy of the six‐RBDs mosaic vaccines, we first constructed a mosaic‐Omicron‐NP including BA.1, BA.2, BA.5, and BA.2.75 RBDs and a mosaic‐D/G‐NP including Delta and D614G RBD respectively (Figure S3A, Supporting Information). The results showed a seesaw effect of the reciprocal antiserum between the early strains and Omicron, suggesting the necessity of combining the six RBDs together (Figure S3A,B, Supporting Information). Notably, as the mosaic‐ODG NP vaccine induced a higher robust neutralization titer with broader cross‐neutralization activity (Figure S3B, Supporting Information), we chose mosaic‐ODG as our final mosaic NP vaccine candidate.

**Figure 2:**
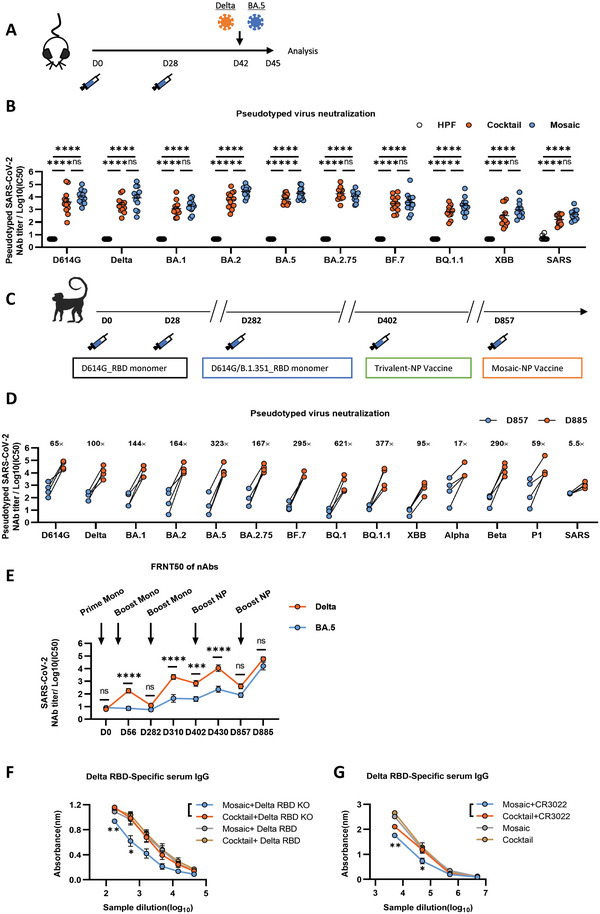
The immunization effectiveness of the mosaic RBD nanoparticle vaccines. A) Immunization schedule. K18‐hACE2 mice were immunized with either 10 µg mosaic RBD NP vaccine, cocktail NP vaccine, or the molar equivalent of Sd‐HPF NP through the subcutaneous route at days 0 and 28. Mice were challenged with authentic SARS‐CoV‐2 (Delta/BA.5) on day 42 and euthanized three days post‐challenge for analysis. B) Pseudovirus neutralization Ab titers of sera from K18‐hACE2 mice immunized with cocktail and mosaic RBD nanoparticles at week 6 against SARS and a panel of nine SARS‐CoV‐2 mutant variants (*n* = 12). C) Immunization schedule. Rhesus Macaques were primed and boosted with D614G_RBD monomer intramuscularly vaccinated at days 0 and 28. On day 282, Rhesus Macaques was vaccinated with the third dose of D614G/B.1.351_RBD monomer. On day 402. the fourth dose of the trivalent RBD‐NP vaccine, which includes the D614G, B.1.351, and Delta individual RBD NPs, was given an intramuscular injection. On day 857, the Rhesus Macaques were immunized with mosaic RBD NP. D) The NAbs titer for pandemic and potential pre‐emergent SARS‐CoV‐2 virus of Rhesus Macaques before and post the boost of mosaic RBD NP vaccine was determined by pseudotyped virus neutralization assay and represented as IC50. Each dot represents serum from one animal (*n* = 4). The fold changes were calculated by comparing the mean value of each group. E) Each Rhesus Macaque serum nAbs titer against authentic Delta and BA.5 variants virus on different days was evaluated using FRNT50. FRNT50 of nAbs of authentic Delta and BA.5 variants were determined by FRNT and plotted as a time‐course curve. F) The binding affinity of nanoparticle‐vaccinated mice serum with Delta RBD or Delta RBD‐KO was analyzed by ELISA. G) Antibody competition experiments of nanoparticle immunized mouse serum with CR3022 were analyzed by ELISA. OD values versus dilution factors are plotted. Experiments were conducted independently in triplicates. Data are represented as mean ± SEM. Adjusted *p*‐values were calculated by one‐way ANOVA with Tukey's multiple comparison test. Asterisks indicate significant differences between groups linked by horizontal lines. *p ≤ 0.05, **p ≤ 0.01, ***p ≤ 0.001, ****p ≤ 0.0001, ns = not significant.

To evaluate the immunogenicity of mosaic versus cocktail NP vaccine, we immunized the transgenic hACE2‐K18 mice through the subcutaneous route at weeks 0 and 4 (**Figure** **2**A). The RBD‐specific IgG titer was high in all NP‐immunized hACE2 mice two weeks after the boost (Figure S4A, Supporting Information). The pseudovirus neutralization results showed that the nAbs induced by both cocktail and mosaic vaccines significantly inhibited all pseudotyped variants, including D614G, Delta, BA.1, BA.2, BA.5, BA.2.75, BF.7, BQ.1.1, XBB and SARS. Notably, the mosaic vaccine exerted significantly higher nAb activity than cocktail antisera against BA.2, BA.5, and BQ.1.1 pseudotyped viruses (Figure 2B). To further evaluate the efficacy of the mosaic NP vaccine in the non‐human primate, we applied a sequential immunization strategy in previously immunized rhesus macaques (Figure 2C).^[^ ^10b^ ^]^ The RBD‐specific IgG titers against the above SARS‐CoV‐2 strains were relatively high after the mosaic NP vaccine booster, reaching ≈10^5^ after the vaccination at day 857 (Figure S5, Supporting Information). The pseudovirus neutralization results showed that, after a new boost with the mosaic NP vaccine, a broader neutralizing activity against major pandemic variants, including BA.2, BA.5, BA.2.75, BF.7, BQ.1.1, BQ.1, and XBB were significantly increased with a range from 95‐ to 621‐fold (Figure 2D). Further, we conducted a focus reduction neutralizing test (FRNT) to test the robustness of the nAbs against the authentic Delta and BA.5 strains in rhesus macaques. Following the mosaic NP vaccine booster injection, the nAb titers against both authentic Delta and BA.5 viruses significantly increased (Figure 2E), indicating that mosaic NP vaccines produced effective protective nAbs in rhesus monkeys.

To evaluate whether our mosaic NP vaccine elicits more broadly cross‐protective class IV antibodies than the cocktail, we examined the antibodies in sera of NP‐vaccinated mice for their binding affinity to Delta RBD or Delta RBD‐KO, which harbors several mutants (S383A, P384A, T385A, and K386A site) and loss the binding affinity to the class IV antibody such as CR3022 but not to the class II antibody such as C144 (Figures S1A and S6A, Supporting Information). We found that the sera of mosaic NP‐vaccinated mice with Delta RBD‐KO binding affinity was significantly reduced than cocktail NP‐vaccinated mice (Figure 2F). In addition, we performed antibody competition experiments for the immunized mouse sera against the CR3022 antibody, which showed that the CR3022 antibody significantly inhibited the binding of sera of mosaic NP‐vaccinated mice to Delta RBD (Figure 2G). Moreover, we found that the percentages of RBD‐specific GC B cells and RBD‐specific memory B cells(MBC)within the spleen were significantly higher in mosaic NP‐vaccinated mice than in cocktail NP‐vaccinated mice (Figure S6B,C, Supporting Information). These results revealed that the mosaic NP‐vaccine elicits more RBD‐specific humoral immune responses and class IV antibodies than a cocktail vaccine in mice, illustrating that the mosaic NP vaccine produces superior broadly neutralizing against variant viral strains.

### 2.2 Intranasal but not Subcutaneous Immunization with Mosaic RBD NP Vaccine Induces Superior IgA and IgG Secretions in the Lung

**Figure 3:**
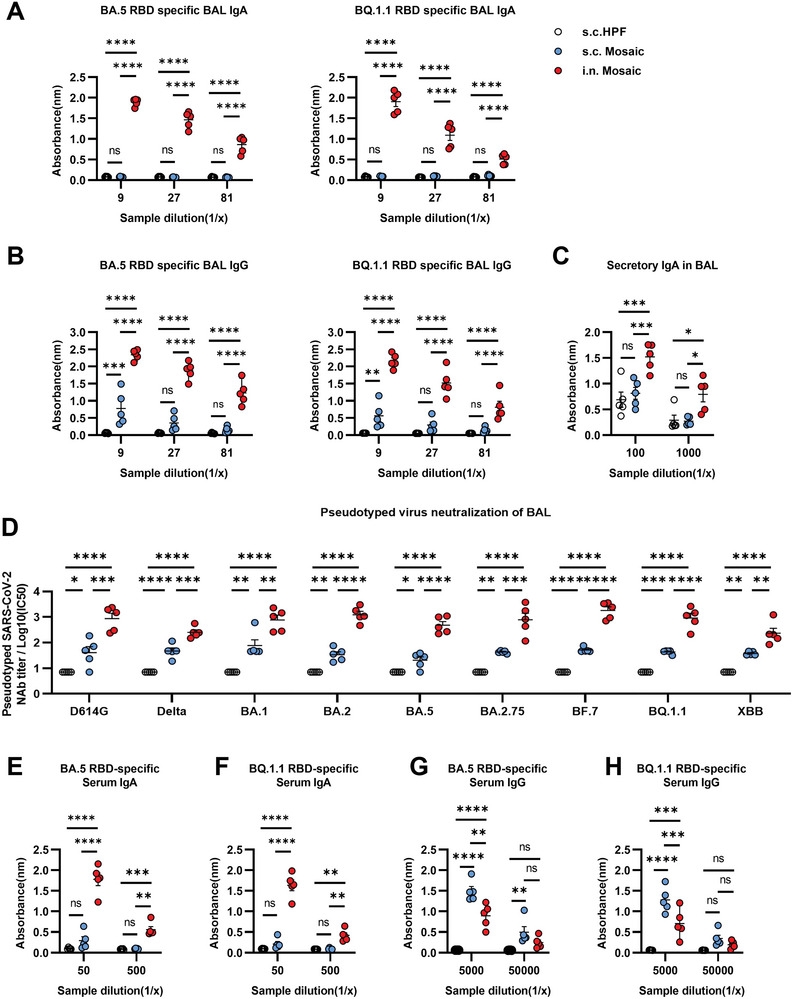
Intranasal but not subcutaneous immunization with mosaic RBD NP vaccine induces potent mucosal immune responses in the lung. A–H) BALB/c mice were vaccinated with either 10 µg mosaic RBD NP vaccine subcutaneously (s.c), intranasally (i.n), or the molar equivalent of Sd‐HPF NP subcutaneously (s.c) at weeks 0 and 4. Vaccinated mice were euthanized for the analysis of immune response at week 6 (*n* = 5). A,B) Levels of BA.5 or BQ.1.1 RBD‐specific IgA and IgG were measured from BAL fluid by enzyme‐linked immunosorbent assay (ELISA). C) Secretory IgA (sIgA) level in the BAL fluid was measured by ELISA. D) Measurement of neutralization titer against pseudotyped SARS‐CoV‐2 virus in BAL fluid. E–H) levels of BA.5 or BQ.1.1 RBD‐specific IgA and IgG from serum were measured by ELISA. Data are represented as mean ± SEM. Adjusted p values were calculated by two‐way ANOVA with Tukey's multiple comparisons test. Asterisks indicate significant differences between groups linked by horizontal lines. *p ≤ 0.05, **p ≤ 0.01, ***p ≤ 0.001, ****p ≤ 0.0001, ns = not significant.

As the current vaccines exhibit low efficacy in preventing infection in the upper respiratory tract, it is important to investigate whether the establishment of mucosal immunity with the NP vaccine could reach efficient protection. To this end, we first compared the immune responses after intranasally or subcutaneously inoculating the mosaic NP vaccine. We collected the bronchoalveolar lavage (BAL) fluid of mice to measure the RBD‐specific IgA and IgG and found that the intranasal immunization induced a higher level of BA.5 or BQ.1.1 RBD‐specific IgA and IgG antibodies in the BAL fluid compared with subcutaneous immunization. Moreover, IgA in the BAL was found only after intranasal immunization (**Figure** **3**A,B). As IgA in local mucosal secretions was in the form of sIgA, we also measured the levels of sIgA in BAL fluid. Consistently, sIgA was mainly produced in response to intranasal immunization with the mosaic NP vaccine (Figure 3C). In addition, we found intranasal vaccination with mosaic vaccine could elicit broadly NAbs in BAL fluid (Figure 3D). Moreover, RBD‐specific serum IgA titer induced by intranasal vaccination was higher than that by subcutaneous immunization (Figure 3E,F), while the RBD‐specific serum IgG titer was lower than that by subcutaneous immunization (Figure 3G,H). These results indicated that intranasal vaccination with the mosaic NP vaccine induced superior IgA and IgG secretions in the lung than subcutaneous immunization.

### 2.3 Intranasal Immunization Induces Memory T Cells, Memory B Cells, and Plasma Cells Resident in Lung Tissue

**Figure 4:**
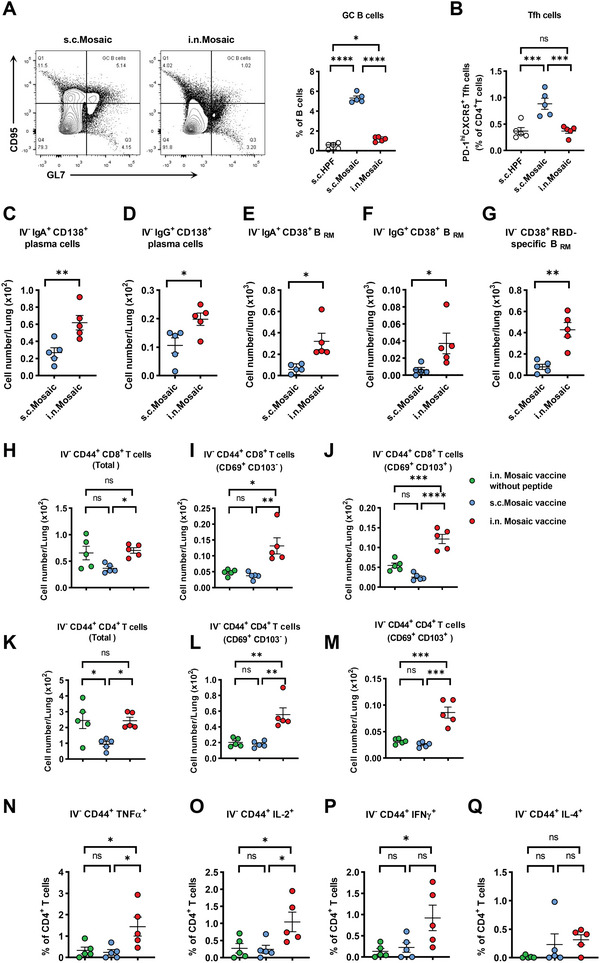
Intranasal immunization induces memory T cells, memory B cells, and plasma cells resident in lung tissue. A) The portions of germinal center B cells and B) T follicular helper cells within the spleen were analyzed by flow cytometry. C–Q) Mice were intravenously injected with anti‐CD45 antibody (2 µg per mouse) 5 min before euthanasia to label circulating immune cells inside the blood vessels (*n* = 5). C–G) various tissue‐resident (IV labeling antibody negative) B cell subsets were measured, including IgA^+^ plasma cells, IgG^+^ plasma cells, IgA^+^ B_RM_ cells, IgG^+^ B_RM_ cells, and RBD‐specific B_RM_ cells in lung tissues from subcutaneous or intranasal immunization mice. H–M) Lung‐tissue‐resident CD8 T cell and CD4 T cell responses from intranasal immunization mosaic Np‐vaccines without six highly conserved T cell epitope peptides, subcutaneous or intranasal immunization mosaic Np‐vaccines mice. Shown are quantification of CD44^+^ CD8 T cells, CD69^+^ CD103^−^ CD44^+^ CD8 T cells, or CD69^+^ CD103^+^ CD44^+^ CD8 T cells, CD44^+^ CD4 T cells, CD69^+^ CD103^−^ CD44^+^ CD4 T cells, or CD69^+^ CD103^+^ CD44^+^ CD4 T cells in lung tissue. N‐Q) Lung cells of vaccinated mice were incubated with mosaic T cell peptides pool, respectively. The percentages of TNF‐α, IL‐2, IFN‐γ, and IL‐4 positive IV‐CD45^−^ CD44^+^ CD4 T cells were determined by ICCS. Experiments were conducted independently in triplicates. Data are represented as mean ± SEM. Adjusted p values were calculated by one‐way ANOVA with Tukey's multiple comparison test. Asterisks indicate significant differences between groups linked by horizontal lines. *p ≤ 0.05, **p ≤ 0.01, ***p ≤ 0.001, ****p ≤ 0.0001, ns = not significant.

We further examined the response of various immune cells in different tissues. We found that the subcutaneous immunization route elicited a significant increase of GC B cells and TFH cells within the spleen than the intranasal vaccination route (**Figure** **4**A,B), indicating that the subcutaneous immunization stimulates a much stronger systemic immune response than intranasal immunization. Conversely, the intranasal vaccination with the mosaic NP vaccine induced higher numbers of plasma cells and tissue‐resident memory B cells (B_RM_) in the lung tissue (Figure 4C–G), as well as higher numbers of class‐switched antibody‐secreting plasma cells expressing IgA or IgG (Figure 4C,D). We also found increased class‐switched B_RM_ cells expressing IgA or IgG in lung tissue (Figure 4E,F). In contrast, subcutaneous immunization failed to generate B_RM_ cells (Figure 4E,F). Additionally, intranasal vaccination with mosaic NP vaccine resulted in significantly higher proportions of RBD‐specific B_RM_ cells than subcutaneous vaccination (Figure 4G). Thus, all these data support that intranasal immunization induces a potent humoral response in the respiratory tissue.

To determine whether the six highly conserved T cell epitope peptides of mosaic NP‐vaccines could induce potent T cell responses, we vaccinated BALB/c mice with mosaic NP‐vaccine without six highly conserved T cell epitope peptides via the intranasal routes at weeks 0 and 4 as control. We found that intranasal immunization induced higher numbers of memory CD8^+^ or CD4^+^ T cells than subcutaneous vaccination in lung tissue (Figure 4H,K). We also found that the intranasal immunization mosaic NP‐vaccine with six highly conserved T cell epitope sequences significantly increased the CD8^+^ T_RM_ cells and CD4^+^ T_RM_ cells in lung tissue (Figure 4I,J,L,M). To further characterize the CD4^+^ T_RM_ cell response, we used a mosaic T cell peptide pool to stimulate the isolated lung lymphocytes of vaccinated mice and analyzed different T cell populations by intracellular cytokine staining (ICCS) assays. We found that the mosaic NP vaccines with six highly conserved T cell epitopes elicited more antigen‐specific CD4^+^ T cells expressing TNF‐a^+^, IL‐2^+^, and IFN‐γ^+^ than the vaccine without six highly conserved T cell epitopes (Figure 4N–P). Further, six highly conserved T cell epitope sequences in mosaic NP vaccines led to a higher number of tissue‐resident CD4^+^ T helper type 1 (TH1) but not TH2 CD4^+^ T cells (Figure 4N–Q).

### 2.4 Protective Efficacy of the Intranasal Immunization with Mosaic Vaccine against Delta and BA.5 Variant Infections in hACE2‐K18 Mice

**Figure 5:**
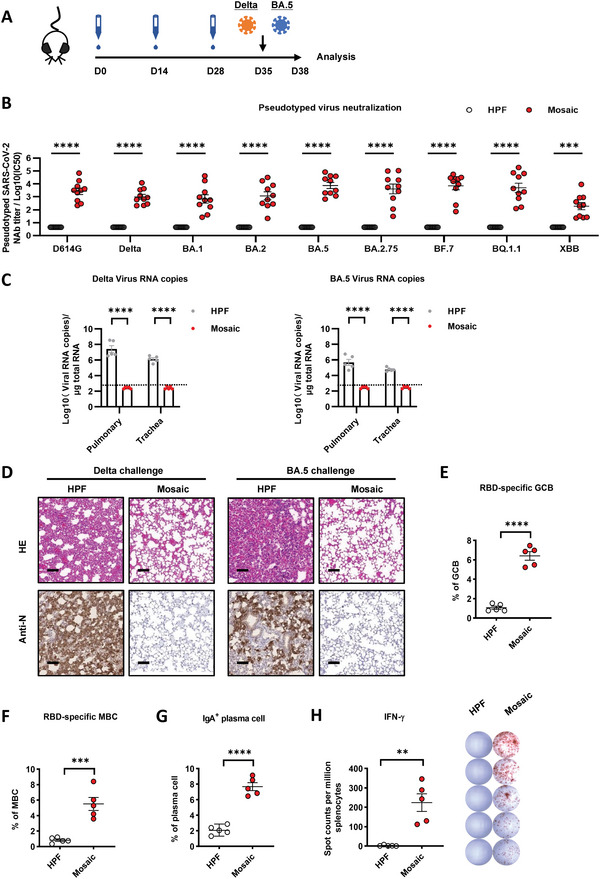
The intranasal immunization with the mosaic vaccine elicited strong humoral immune responses to protect against Delta and BA.5 variant infections in hACE2‐K18 mice. A) Immunization schedule. K18‐hACE2 mice were unadjuvanted immunized with either 10 µg mosaic RBD NP vaccine or the molar equivalent of Sd‐HPF NP through the intranasal route at days 0, 14, and 28. The vaccinated mice were challenged with authentic Delta and BA.5 variants at day 35 and euthanized three days post‐challenge for analysis. B) Pseudovirus neutralization Ab titers of mosaic RBD nanoparticle elicited sera from K18‐hACE2 mice at day 35 against a panel of 9 SARS‐CoV‐2 mutant variants (*n* = 10). C) Viral RNA copies in the lungs and trachea of each mouse were determined by qRT‐PCR and plotted as log_10_ copies per microgram. The dotted line indicates the limit of detection (LOD). Each dot represents a tissue sample from one animal (*n* = 5). D) H&E staining and immunohistochemistry (IHC) against N proteins were evaluated in the lungs of mice. Scale bars represented 100 µm. E–H) Mice were euthanized for the analysis of immune response on day 35. The percentages of E) RBD‐Specific‐GC B cells (CD19^+^ CD95^+^ GL7^+^ RBD‐BV421^+^), F) RBD‐Specific MBC (CD19^+^ IgD^−^ CD38^+^ RBD‐BV421^+^), and G) IgA^+^ plasma cells (CD19^−^ IgD^−^ CD138^+^ IgA^+^) within the spleen of each vaccine group were determined by FCM (*n* = 5). Antigen‐specific cells were analyzed by BV421‐conjugated BA.2 RBD probe. H) ELISpot assays were conducted for IFN‐γ secretion in splenocytes. Data are represented as mean ± SEM. Adjusted p values were calculated by one‐way ANOVA with Tukey's multiple comparison test. Asterisks indicate significant differences between groups linked by horizontal lines. *p ≤ 0.05, **p ≤ 0.01, ***p ≤ 0.001, ****p ≤ 0.0001, ns = not significant.

Finally, we evaluated the protective efficacy of unadjuvanted intranasal vaccination with the mosaic NP vaccine against Delta and BA.5 variant infections in hACE2‐K18 mice (**Figure** **5**A). The intranasal immunization with the mosaic NP vaccine induced a robust RBD‐specific IgG titer and broadly neutralizing antibodies in hACE2‐K18 vaccinated mice sera (Figure S7A, Supporting Information) (Figure 5B). The vaccinated hACE2‐K18 were challenged intranasally with authentic Delta and BA.5 variants, respectively, and the mice were euthanized 3 days post‐challenge for analysis. While the viral RNAs in the lungs and tracheas in control hACE2 mice had an average of 1.33 × 10^8^ and 1.74 × 10^6^ copies for Delta and 1.77 × 10^6^ and 6.93 × 10^4^ copies per µg total RNA for BA.5 strains, respectively, the mosaic NP vaccinated hACE2 mice exhibited undetectable levels of viral RNA (Figure 5C). Moreover, a significant reduction of inflammation in vaccine‐immunized hACE2 mice was also observed. The SARS‐CoV‐2 nucleocapsid (N) antigen was undetectable in all nanoparticle‐vaccinated hACE2 mice but was detected in the unvaccinated mice (Figure 5D). Similarly, hACE2 mice subcutaneously immunized with the mosaic NP vaccine had undetectable levels of viral RNA in the lungs and tracheas (Figure S4B, Supporting Information), and the SARS‐CoV‐2 N antigen was also undetectable in lung tissue (Figure S4C, Supporting Information). Meanwhile, we found that mosaic NP vaccines elicited a significant increase of RBD‐specific GC B cells, memory B cells, IgA^+^ plasma cells, and IFN‐γ^+^ T‐cells in the spleen (Figure 5E–H). The results further indicated that the unadjuvanted intranasal immunization with the mosaic NP vaccine induced robust humoral and cellular immune responses against SARS‐CoV‐2 variants and caused complete protection against the infection of authentic Delta or BA.5 strains in the hACE2‐K18 transgene mice.

## 3 Discussion

NP vaccines are of the advantages of high potency, low side effects, and low production and storage costs.^[^ ^13^ ^]^ Due to the similar size as natural viruses, the nanoparticle vaccines can be easily captured by antigen‐presentation cells and B‐cells with antigen‐specific BCRs.^[^ ^14^ ^]^ Such an adaptive design supplies an excellent platform for developing next‐generation vaccines as they provide a pathway to induce robust neutralizing antibody production.^[^ ^15^ ^]^ Importantly, this technical route can display various antigens on the surface of a nanoparticle simultaneously to form a mosaic vaccine.^[^ ^8a^ ^]^ Thus, the antigens from various viral substrains are presented and presented to a B cell at the same time.^[^ ^8^, ^16^ ^]^ The common epitope in various antigens can strongly stimulate the same BCR simultaneously, resulting in the amplified B cell clones that produce broad‐spectrum neutralizing antibodies.^[^ ^8^, ^16^ ^]^ The development of this nanoparticle‐based mosaic vaccine to induce broadly neutralizing antibodies is expected to be a powerful tool against current and future variants of sarbecoviruses. In this study, we first selected four subtypes of Omicron RBD, including BA.1, BA.2, BA.5, BA.2.75, Delta, and the original strains (D614G)RBD as the mosaic vaccine antigens, which ensure maximum protection against Omicron and its subvariants. The antigen combination of our mosaic vaccines offers the best broad‐spectrum while retaining maximum specificity for currently circulating strains and their following lineages, such as BF.7, BQ.1, and XBB. In addition, this mosaic NP vaccine contains six highly conserved T cell epitopes and elicits a robust cellular immune response. Taken together, we have developed a new universal SARS‐CoV‐2 vaccine against the newly‐emerged sublineages of SARS‐CoV‐2, as well as other sarbecoviruses. To further evaluate the efficacy of the mosaic NP vaccine in non‐human primates, we utilized a sequential immunization strategy in rhesus macaques previously immunized.^[^ ^10b^ ^]^ These monkeys received multiple rounds of immunizations with the RBD monomer vaccine to mimic the sequential immunization strategy, which simulates the multiple‐round inoculations in humans. We employed the same group of monkeys to evaluate the efficacy of the mosaic vaccine, administering the immunization on day 857 after a gap of 455 days. Following the booster injection of the mosaic NP vaccine, the neutralizing antibody (nAb) titers against both Delta and BA.5 viruses showed a significant increase. These results indicate that the mosaic vaccine holds tremendous potential as a universal solution against SARS‐CoV‐2 and other sarbecoviruses.

Although the population worldwide has been widely vaccinated with various vaccines based on the early strains of SARS‐CoV‐2,^[^ ^17^ ^]^ the trend of vaccines “preventing severe disease, but not preventing infection” is quite obvious with the continuous emergence of SARS‐CoV‐2 variants.^[^ ^18^ ^]^ Mucosal immunity provides the first line of defense against various pathogens at their entry site. Since almost all the current COVID‐19 vaccines are administered intramuscularly, their effect in inducing mucosal immunity is lower than in the recovered patients, while the antibody response to the vaccinated recipients' peripheral blood was significant.^[^ ^7^ ^]^ In addition, compared with recovered patients, vaccine recipients had few S protein‐specific T cells and B cells in BAL.^[^ ^7a^ ^]^ In this study, we found intranasal vaccination with the mosaic vaccine induced superior neutralizing IgA and IgG secretion in the lung than that by subcutaneous immunization in mice. Besides, the cellular immune response against the conserved T‐reactive epitope peptides has been induced. Importantly, these mucosal immune responses are characterized by the increased percentages of tissue‐resident RBD‐specific memory B cells, plasma cells, and tissue memory T cells, which is significantly higher than that vaccinated subcutaneously. These results suggested that mucosal immune memory has been successfully established. Our work expands the capability of self‐assembled NP vaccines. Without any adjuvants, our mosaic NP vaccine potently elicits mucosal immunity with significant cross‐protection activity and merits being further developed as a new universal vaccine against SARS‐CoV‐2 and other sarbecoviruses.

## 4 Conclusion

The mucosal immune system provides essential protection against respiratory pathogens at the very beginning and almost all the current major COVID‐19 vaccines are administered intramuscularly, which cannot induce effective mucosal immunity. It is necessary and urgent to develop a universal vaccine preventing infection in the upper respiratory tract. Here, we designed a mosaic RBD NP vaccine, which can induce potent humoral and cellular immune responses and strong mucosal immunity with potent broad cross‐protective activity, shedding light on overcoming the immune evasion caused by severe mutation of the SARS‐CoV‐2 variants and blocking the viral invasion at the upper respiratory tract. Our work expands the capability of self‐assembled NP vaccines. Without adjuvants, our mosaic NP vaccine potently elicits mucosal immunity with significant cross‐protection activity and merits being further developed as a new universal vaccine against SARS‐CoV‐2 and other sarbecoviruses.

## 5 Experimental Section

### Ethics Statements

The Ethics Review Board of Sun Yat‐sen University approved this study. All experiments on mice in the research protocol were approved by the Ethics Committee of Zhongshan School of Medicine (ZSSOM) of Sun Yat‐sen University on Laboratory Animal Care (Assurance Number: 2017–061) and were performed following relevant institutional and national guidelines and regulations. Non‐human primate experiments were approved by the Institutional Animal Care and Use Committee (IACUC) of Guangdong Landau Biotechnology Co, Ltd. (IACUC Approval No: LDACU 20200216‐01).

### Cells and Viruses

HEK293T and Vero E6 cells were obtained from ATCC. These adherent cells were cultured in DMEM supplemented with 1% penicillin‐streptomycin (ThermoFisher) and 10% FBS (ThermoFisher). HEK293T expressing hACE2 (hACE2/HEK293T) was constructed. FreeStyle 293F suspension cells were stored in Union 293F (Union) containing 8 mM glutamine (ThermoFisher), and 1% penicillin‐streptomycin (ThermoFisher) and placed on a polycarbonate vented conical flask shaker at 37 °C, 8% CO_2_, and a shaker at 120 rpm. All cells were regularly tested for mycoplasma DNA using PCR, and it confirmed that they were not mycoplasma‐positive. The authentic GDPCC‐nCoV‐ Delta‐ and GDPCC‐nCoV‐Omicron BA.5‐ related experiments, which were isolated and authorized by Guangdong Center for Disease Control, were carried out in the BSL‐3 facility at Sun Yat‐sen University.

### Animal Models

A total of 56 K18‐hACE2 transgenic mice of both genders (C57BL/6 strain) were procured from GemPharmatech Co, Ltd. Additionally, 60 male BALB/c mice aged 5–6 weeks, reared in a specific‐pathogen‐free (SPF) environment, were obtained from Guangdong Medical Laboratory Animal Center. The number of mice used for the C57BL/6 and BALB/c strains is indicated in the figure legend. All mice were raised and vaccinated in the SPF facilities at the Laboratory Animal Center of Sun Yat‐Sen University. Two male and two female adult Rhesus Macaques between 2 and 4 years old were purchased previously from Guangdong Landau Biotechnology Co, Ltd. Monkeys experiments were conducted according to the guidelines and regulations of the Laboratory Monitoring Committee of Guangdong Province of China.

### Protein Expression and Purification

The construction of the RBD nanoparticle vaccine was described above. To further improve the binding efficiency of the NP vaccine, the Gv/Sd connection system was used to enhance the efficiency and production of the SARS‐CoV‐2 NP vaccine. Sd‐HPF could be self‐assembled to be a 24‐mer nanoparticle and conjugated with GV‐RBD robustly. Sd‐HPF was expressed and purified from BL21 (Takara) prokaryotic expression induced by Isopropyl thio‐β‐galactoside (IPTG). The bacterial cultures were harvested and lysed in Tris buffer (20 mM Tris, 50 mM NaCl, pH 7.5). The lysate supernatants were heated to 70 °C for 15 min to precipitate most of the *Escherichia coli* proteins. After centrifugation and concentration, the supernatants were loaded onto a Superose 6 Increase (GE Healthcare) size exclusion column and eluted with the Tris buffer at a rate of 0.5 ml min^−1^. The elution volume of Sd‐HPF NP was 14 ml. The concentration of Sd‐ferritin was determined by the BCA assay method. The bacterial endotoxins in nanoparticles were quantified by the *Tachypleus* amebocyte lysate test (≤ 10 EU per dose). The sequences of BA.1_RBD‐N395‐403, BA.2_RBD‐N316‐333, BA.5_RBD‐N360‐370, BA.2.75_RBD‐S539‐546, Delta _RBD‐S1136‐1155, D614G_RBD‐S865‐873, BF.7_RBD, BQ.1.1_RBD, XBB_RBD, SARS_RBD, Delta S6P, and BA.2 S6P were cloned into pcDNA3.1 vector, expressed and purified from Expi293Fcells. The RBD was genetically fused at the N‐terminus of GV‐Tag, which could be conjugated with SD‐ferritin in the manner of an isopeptide bond,^[^ ^19^ ^]^ followed by transfection and purification with the Ni‐affinity chromatography. All purified proteins were confirmed with gel electrophoresis of SDS‐PAGE, together with the Coomassie blue staining.

### Animal Vaccination

For the subcutaneous vaccination route, each group of BALB/c or K18‐ACE2 mice was subcutaneously immunized with either 10 µg mosaic RBD NP vaccine, cocktail NP vaccine, or the molar equivalent of Sd‐HPF NP with Alum‐adjuvanted at days 0 and 28, respectively. For the intranasal vaccination route, K18‐hACE2 mice were unadjuvanted immunized with either 10 µg mosaic RBD NP vaccine or the molar equivalent of Sd‐HPF NP through the intranasal route at days 0, 14, and 28. The hACE2‐K18 mice were challenged with the authentic SARS‐CoV‐2 at day 35 after the prime vaccination and were euthanized 3 d post‐infection for analysis. For the Rhesus monkey vaccination, four monkeys were initially primed and boosted with D614G_RBD monomer at days 0 and 28; On day 282, Rhesus Macaques were vaccinated with the third dose of D614G/B.1.351_RBD monomer and followed by 50 µg doses of trivalent RBD‐NP vaccine on day 402.^[^ ^10b^ ^]^ At day 857 after initial immunization, Rhesus Macaques were inoculated with 50 µg of mosaic RBD NP vaccine. The vaccines were formulated with an equal volume of Alum (InvivoGen) adjuvant through the intramuscularly vaccinated route for Rhesus monkeys. Four weeks later, on day 885, sera were collected from the Rhesus monkeys for pseudotyped virus neutralization assays and FRNT50 testing.

### SARS‐CoV‐2 Infection

Transgenic K18‐hACE2 mice, immunized with indicated vaccines, were challenged with authentic SARS‐CoV‐2 Delta (B.1.617.2) or Omicron BA.5 strains in the BSL‐3 facility. Mice were anesthetized with isoflurane and inoculated intranasally with 1 × 10^5^ FFU of SARS‐CoV‐2 viruses. The lungs were collected at 3 days post‐infection (d.p.i.).

### Quantitative Reverse Transcription Polymerase Chain Reaction (qRT‐PCR)

The trachea and lung of challenged hACE2‐K18 mice were collected and homogenized with gentleMACS M tubes (Miltenyi Biotec, 130‐093‐236) in a gentle‐MACS dissociator (Miltenyi Biotec, 130‐093‐235). RNAs were extracted using RNeasy Mini Kit (QIAGEN, 4104) according to the manufacturer's instruction, followed by the qRT‐PCR to determine the viral RNA copies of different tissues utilizing a one‐step SARS‐CoV‐2 RNA detection kit (PCR‐Fluorescence Probing) (DaAn Gene Co., DA0931). The SARS‐CoV‐2 nucleocapsid (N) gene was cloned into a pcDNA3.1 expression plasmid for standards to generate a standard curve. The indicated copies of N standards were tenfold serially diluted from 10^9^ to 10^2^ and proceeded to qRT‐PCR utilizing the same one‐step SARS‐CoV‐2 RNA detection kit to obtain standard curves. The reactions were carried out on a BioRad CFX according to the manufacturer's instructions. The viral RNA copies of each tissue were calculated into copies per µg total RNA and presented as a log_10_ scale.

### Histopathology and Immunohistochemistry

The K18‐hACE2 mice challenged with SARS‐CoV‐2 were euthanized in the BSL‐3 facility. Lungs were collected and fixed in 4% paraformaldehyde buffer for 48 h at 4 ˚C, followed by embedding with paraffin. Longitudinal sections were performed on these tissues. The sections (3–4 µm) were stained with hematoxylin and eosin (H&E). For immunohistochemistry, lung sections of each mouse were incubated with rabbit anti‐SARS‐CoV‐2 Nucleoprotein (N) at 1:1000 dilution, and the IHC was conducted as published before.^[^ ^20^ ^]^

### Enzyme‐Linked Immunosorbent Assay (ELISA)

The six recombinant Gv_RBD proteins were diluted to 5 µg mL^−1^ with coating buffer and coated on high‐binding 96‐well plates (Corning) overnight at 4 °C, respectively. After washing with PBS three times, the plates were blocked with 5% non‐fat milk/PBS for 1 h at room temperature. The plates were washed three times with PBS/T (containing 1% Tween‐20) again, and the animal serum or BALF was diluted serially in PBS, followed by incubating the plates for 1 h at 37 °C. After washing with PBS/T, HRP‐conjugated goat anti‐mouse or goat anti‐monkey secondary antibody (Invitrogen) was added at a dilution of 1:4000 to detect antigen‐specific lgG antibody in serum of BALB/c, K18 mice, or rhesus macaques. Correspondingly, the antigen‐specific IgA antibody in BALF was detected by adding HRP‐conjugated goat anti‐mouse secondary antibody with a dilution of 1:1000. After incubating for another 1 h, the plates were washed with PBS/T. Subsequently, 50 µL HRP substrate TMB solution (eBioscience) per well was added under dark, and the reaction was terminated with stop solution (Solarbio) after sufficient development. The absorption was measured at 450 nm. GraphPad Prism 8.0 software was used to perform non‐linear regression analysis on the data to calculate the endpoint titer.

### Pseudotyped Virus Neutralization Assay

The generation protocol was described as published before.^[^ ^4e^ ^]^ In brief, the plasmids expressing the respective mutant pseudotyped virus spike protein of D614G, Delta, BA.1, BA.2, BA.5, BA.2.75, BF.7, BQ.1.1, BQ.1, XBB, and SARS were constructed. HEK293T cells were co‐transfected with the psPAX2 (Addgene) plasmid, the lentiviral plasmid expressing luciferase (Addgene), and the plasmid expressing the respective mutant spike proteins by using polyethyleneimine (PEI, Sigma). 48 h after transfection, the culture supernatant was collected and filtered with a 0.20 µm filter and then stored at −80 °C. Virus titration was performed by serially diluting the viral infection of hACE2‐293T cells, and the infectivity was measured by detecting luminescence. The serum of all immunized animals was serially diluted and incubated with a pre‐titrated amount of pseudotyped SARS‐CoV‐2 virus at 37 °C for 1 h. Subsequently, the serum/virus mixture was added to the wells containing 2 × 10^4^ hACE2‐293T cells and incubated at 37 °C in 5% CO2 for 48 h. Then the cells were lysed with lysis buffer (Promega), and the lysate was measured by detecting the relative luminescence unit (RLU) in the photometer (Promega) to measure the luciferase activity. GraphPad Prism 8.0 software was used to analyze the serum‐neutralizing antibody titers of the pseudotyped virus.

### Size‐Exclusion Chromatography (SEC)

The six Gv_RBD proteins were incubated with excessive Sd‐Ferritin nanoparticles in the Tris‐HCl buffer overnight at 4 °C, respectively. The Gv/SD‐conjugated protein was verified by size‐exclusion chromatography (SEC). The conjugated protein was subjected to Superose 6 Increase 10/300 (GE Healthcare) size exclusion column at a rate of 0.8 mL min^−1^. The proteins were eluted in retention of 11 mL to 14 ml.

### Cell Isolation and Flow Cytometry

Mice were intravenously injected with anti‐CD45 antibody (2 µg per mouse) 5 min before euthanasia to label circulating immune cells inside the blood vessel. Lung tissue was collected and homogenized with gentleMACS M tubes (Miltenyi Biotec, 130‐093‐236) in a gentle‐MACS dissociator (Miltenyi Biotec, 130‐093‐235) and dissociated through a 70 µm filter. Lung lymphocytes were isolated by means of the Percoll gradient. The spleen was collected with PBS and homogenized through a 70 µm strainer. The red blood cells (RBCs) were removed by adding ACK lysis buffer, followed by centrifuging to discard the supernatant and resuspended spleen cells with PBS. After passing through a 40 µm strainer to obtain single splenocytes, the cells were stained with fluorochrome‐conjugated monoclonal antibodies for 20 min within PBS containing 0.5% BSA on ice. LIVE/DEAD Fixable Viability Dyes (Thermo) were used to gate live cells. The following indicated antibodies were used: CD19‐BV510 (Biolegend 115546), CD38‐AF647 (Biolegend 102716), IgG1‐PerCP/Cyanine5.5 (Biolegend 406612), IgA‐FITC(BD 559354), and BA.2 RBD probe (BV421), IgD‐APC (Biolegend 405713), CD95‐PE‐Cy7 (Biolegend 152617), GL7‐FITC (Biolegend 144604), CD138‐BV421 (Biolegend 142507), CD3‐ PerCP‐Cy5.5 (Biolegend 100328), CD44‐FITC (Biolegend 103021), CD62L‐APC (Biolegend 104411), CD69‐PE‐Cy7 (Biolegend 104511), and CD103‐BV421(Biolegend 121421). The gating strategies for splenocytes are described in Figure S8, Supporting Information.

### Intracellular Cytokine Staining (ICCS)

Splenocytes were isolated from the vaccinated mice and seeded into 24‐well plates to quantify the percentages of antigen‐specific T cells; ≈10^6^ cells were added per well. Subsequently, stimulated the cells with mosaic peptides pool (GenScript) at a final concentration of 2 g mL^−1^ and co‐stimulated with 2 µg mL^−1^ anti‐CD28 (1000× Biolegend 102116) at 37 °C with 5% CO_2_ for 1 h. DMSO was used as a negative control, and PMA/ionomycin was used as a positive control. Splenocytes were then blocked with 5 µg mL^−1^ brefeldin A (TargetMOL T6062/20350156) and 2 µM monensin (TargetMOL T1033/22373780) for 5 h to prevent cytokines from passing through the cell membrane. Then the cells were extracellularly stained with fluorochrome‐conjugated monoclonal antibodies for 20 min within PBS containing 0.5% BSA on ice. The following antibodies were used: FVD (Invitrogen 65‐0865‐14), CD3‐ PerCP‐Cy5.5 (Biolegend 100328), CD4‐PE‐Cy7 (Biolegend 566939), CD44‐FITC (Biolegend 103021), followed by treatment of 200 µL IC Fixation buffer (BD Biosciences) overnight at 4 °C. The next day, cells were performed with the 1 mL Permeabilization buffer (Invitrogen 00‐8333‐56). Then the antibodies IL‐2‐BV421(Biolegend 503820), TNF‐α‐BV510 (Biolegend 506339), IFN‐γ‐APC (Biolegend 505810), IL‐4‐PE (Biolegend 504104) were used for the intracellular staining and the flow cytometry analysis.

### Surface Plasmon Resonance (SPR)

The measurements of BA.1_RBD‐N395‐403‐NP, BA.2_RBD‐N316‐333‐NP, BA.5_RBD‐N360‐370‐NP, BA.2.75_RBD‐S539‐546‐NP, Delta _RBD‐S1136‐1155NP, D614G_RBD‐S865‐873‐NP, and Mosaic_RBD‐NP binding to hACE2 were carried out with a BIAcoreT100 instrument (GE Healthcare). The BIAcore CM5 sensor chip and the amine‐coupling kit were purchased from GE Healthcare. hACE2 was attached to a CM5 sensor chip (carboxymethylated dextran covalently attached to a gold surface) with an amine coupling kit (GE Healthcare). BA.1_RBD‐N395‐403‐NP, BA.2_RBD‐N316‐333‐NP, BA.5_RBD‐N360‐370‐NP, BA.2.75_RBD‐S539‐546‐NP, Delta _RBD‐S1136‐1155‐NP, D614G_RBD‐S865‐873‐NP, and Mosaic_RBD‐NP were diluted to different concentrations before injection (30 µL min^−1^). Each protein was monitored for about 120 s of hACE2‐binding protein. The running buffer was cycled with a dissociation time of 200 s. The Biacore T100 instrument was used to record the signal according to standard protocol.

### Focus Reduction Neutralizing Test (FRNT)

The generation protocol was described as published before.^[^ ^10a^ ^]^ In short, the Vero E6 cells were inoculated in 96‐well plates with a density of 2 × 10^4^ cells per well and incubated until the cells reached 90–100% fusion. The serum of rhesus macaques at each time point was continuously diluted 10‐fold. 500 FFU of authentic SARS‐CoV‐2 virus was mixed with diluted serum at 1: 1 and incubated at 37 °C for 1 h. The cell culture medium in the 96‐well plate was discarded and incubated with the virus/serum mixture at 37 °C for 1 h. After the supernatant was discarded, DMEM containing 1.6% CMC was added to each well and incubated for 24 h. The supernatant was discarded the next day, and cells were fixed with 200 µL 4% paraformaldehyde per well. After incubation at 4 °C for 12 h, each plate was disposed of fixed solution and washed with 200 ml PBS three times. Then 100 µL PBS containing 0.2% Triton X‐100 and 1% BSA was added to each well. After reaction at 37 °C for 30 min, each well was washed with 200 µL PBS three times and incubated with 50 µL primary antibody against SARS‐CoV‐2 nucleocapsid (N) (Sino Biological), which was diluted to 1:1000 by PBS containing 1% BSA at 37 °C for 1 h. After incubation with the primary antibody, the cells in each well were washed with 200 µL PBS/T (0.1% Tween) three times. The HRP‐conjugated secondary antibody against rabbit IgG (Sino Biological) was diluted with PBS containing 1% BSA to 1:2000. Fifty µL diluted secondary antibody was added to each well, incubated at 37 °C for 1 h, and washed with PBS/T three times. Then, 50 µL TrueBlue (KPL) was added to each well and incubated at 37 °C for 5 min. After the supernatant was discarded, the plates were washed with ddH2O twice. Residual ddH2O was removed and spot counting was performed by ImmunoSpot Microanalyzer. Reduction rates of the serial dilution assay were analyzed by GraphPad Prism 8.0, and the FRNT50 titer was measured by non‐linear regression.

### IFN‐γ Elispot

Cells were cultured in a mosaic T cell peptide pool (GenScript) at a concentration of 2 µg per well for 18–24 h. The mouse IFN‐γ ELISPOT kit (Dakewe) was used to detect antigen‐specific cells of BALB/c mice according to the manufacturer's protocol. Antigen‐specific spots were then counted by the S6 ultra immunostain reader (Cellular Technology Ltd.), and the number of IFN‐γ‐ positive T cells was counted using ImmunoSpot 5.1.34 software (Cellular Technology Ltd.). The number of spots was converted to the number of spots per million cells and plotted as mean ±SEM.

### Statistical Analysis

The statistical details of the specific experiment, including the statistical test used, number of samples, mean values, SEM, and *p*‐values, were described in the figure legends. For comparison between the two treatments, a student's *t*‐test was used. For comparison between each group with the mean of every other group within a dataset containing more than two groups, one‐way ANOVA with Tukey's multiple comparison test was used. For the immunized mouse BAL associate experiment, two‐way ANOVA with Tukey's multiple comparison test was used. *p ≤ 0.05, **p ≤ 0.01, ***p ≤ 0.001, ****p ≤ 0.0001, ns = not significant. Statistical analyses were conducted utilizing GraphPad Prism 8.0.

## Conflict of Interest

The authors declare no conflict of interest.

## Supporting information

Supporting Information

## Data Availability

The data that support the findings of this study are available from the corresponding author upon reasonable request.
