# Supplementary material for: A Mosaic Nanoparticle Vaccine Elicits Potent Mucosal Immune Response with Significant Cross‐Protection Activity against Multiple SARS‐CoV‐2 Sublineages
Source: Adv Sci (Weinh). 2023 Aug 1;10(27):2301034. doi: 10.1002/advs.202301034 (PMC10520630; doi:10.1002/advs.202301034)
Supplement: Supplementary file 1 — Supporting Information [file ADVS-10-2301034-s001.pdf]

ADVANCED SCIENCE
Open Access

Supporting Information

for *Adv. Sci.*, DOI 10.1002/advs.202301034

A Mosaic Nanoparticle Vaccine Elicits Potent Mucosal Immune Response with Significant Cross-Protection Activity against Multiple SARS-CoV-2 Sublineages

Xiantao Zhang, Shijian Wu, Jie Liu, Ran Chen, Yongli Zhang, Yingtong Lin, Zhihui Xi, Jieyi Deng, Zeyu Pu, Chaofeng Liang, Jinzhu Feng, Rong Li, Keming Lin, Mo Zhou, Yingying Liu, Xu Zhang, Bingfeng Liu, Yiwen Zhang, Xin He* and Hui Zhang**

Supplementary Materials

Figure legend

fig. S1

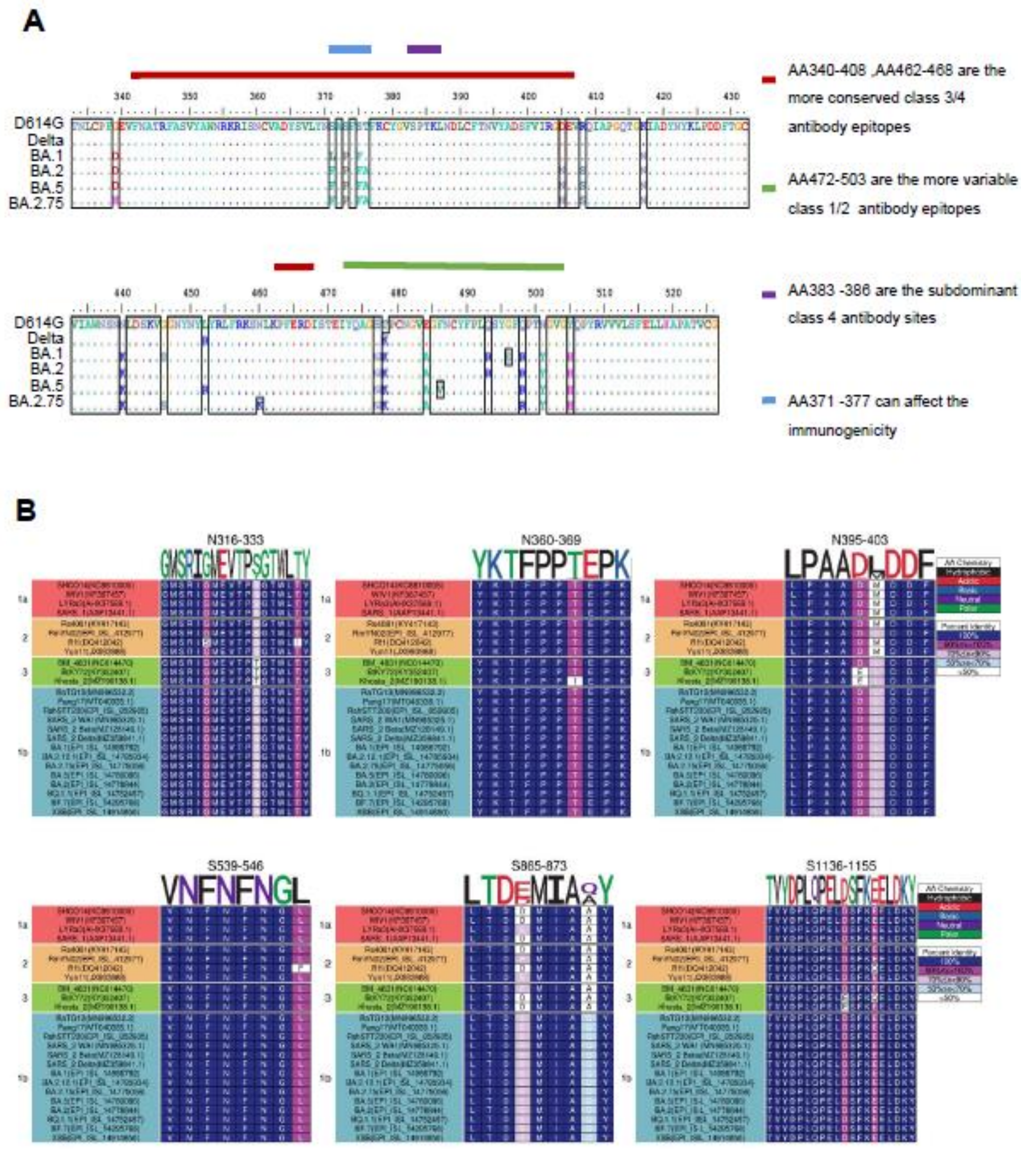

Figure S1 Antigen sequence alignment analysis. (A) Sequence alignment of six mutant strains of SARS-CoV-2 RBD on the Mosaic vaccine. The alignments of sequences were built by using ClustalW method. **(B)** Sequence alignment of six T cell epitope peptides on the N and S proteins of SARS-CoV-2 among Sarbecovirus which contained four clade 1a of Sarbecovirus (SHCO14, WIVI, LYRa3, SARS-CoV-1), four clade 2 of Sarbecovirus (Rs4081, RmYN02, Rf1, Yun11), three clade 3 of Sarbecovirus

(BM-4831, BtKY72, Khosta2), and fourteen clade 1b of Sarbecovirus coronaviruses (RaTG13, Pang17, RshSTT200, SARS-CoV-2 WA1, Beta, Delta, BA.1, BA.2, BA.5, BA.2.75, BA.2.12.1, BQ.1.1, BF.7, XBB). This analysis was performed using MAFFT v7 with a BLOSUM62 scoring matrix and the L-INS-I algorithm. Single-letter abbreviations for amino acids: A, Ala; C, Cys; D, Asp; E, Glu; F, Phe; G, Gly; H, His; I, Ile; K, Lys; L, Leu; M, Met; N, Asn; P, Pro; Q, Gln; R, Arg; S, Ser; T, Thr; V, Val; W, Trp; and Y, Tyr)

fig. S2

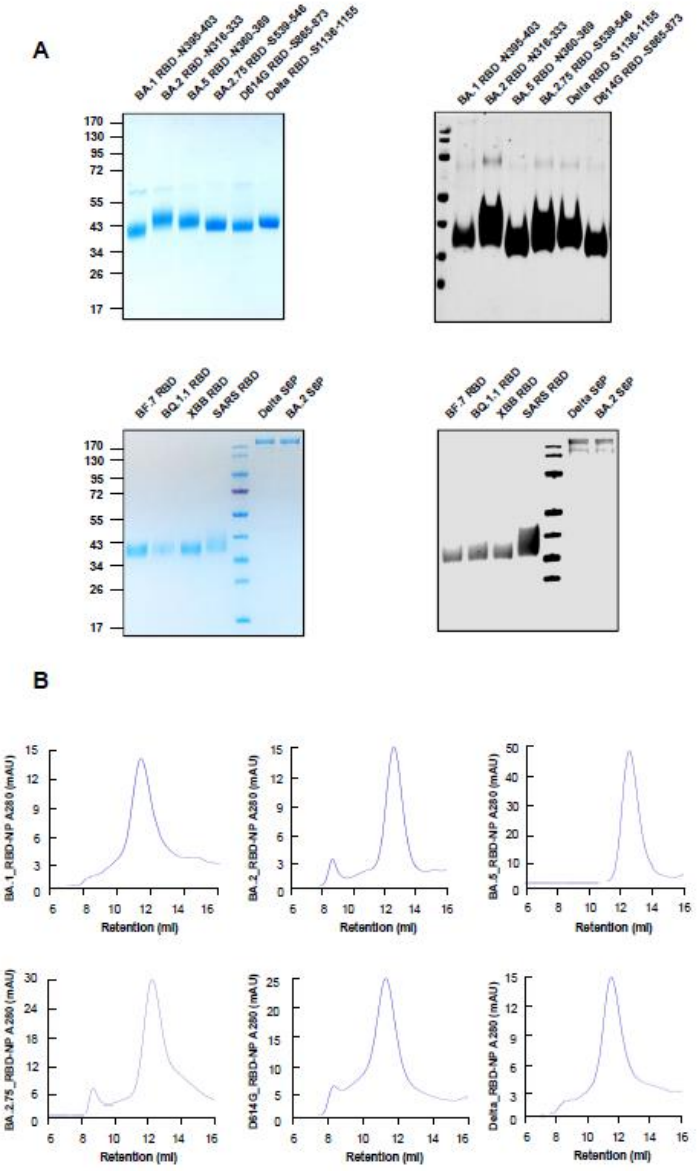

Figure S2 Verification of protein antigen and construction of Mosaic RBD

nanoparticles. (A) Coomassie blue staining (left) of BA.1 RBD-N395-403, BA.2 RBD-N316-333, BA.5 RBD-N360-369, BA.2.75 RBD-S539-546, D614G RBD-S865-873, Delta RBD-S1136-1155, BF.7 RBD, BQ.1.1 RBD, XBB RBD, SARS RBD, Delta S6P, and BA.2 S6P protein antigen . The expression and purity of each protein were confirmed by western blotting with RBD antibodies (right). **(B)** The size exclusion chromatography of BA.1 RBD-N395-403-NP, BA.2 RBD-N316-333-NP, BA.5 RBD-N360-369-NP, BA.2.75 RBD-S539-546-NP, D614G RBD-S865-873-NP, and Delta RBD-S1136-1155-NP. The ultraviolet absorptions at 280 were shown. The retention volume represented the peak of each nanoparticle.

fig. S3

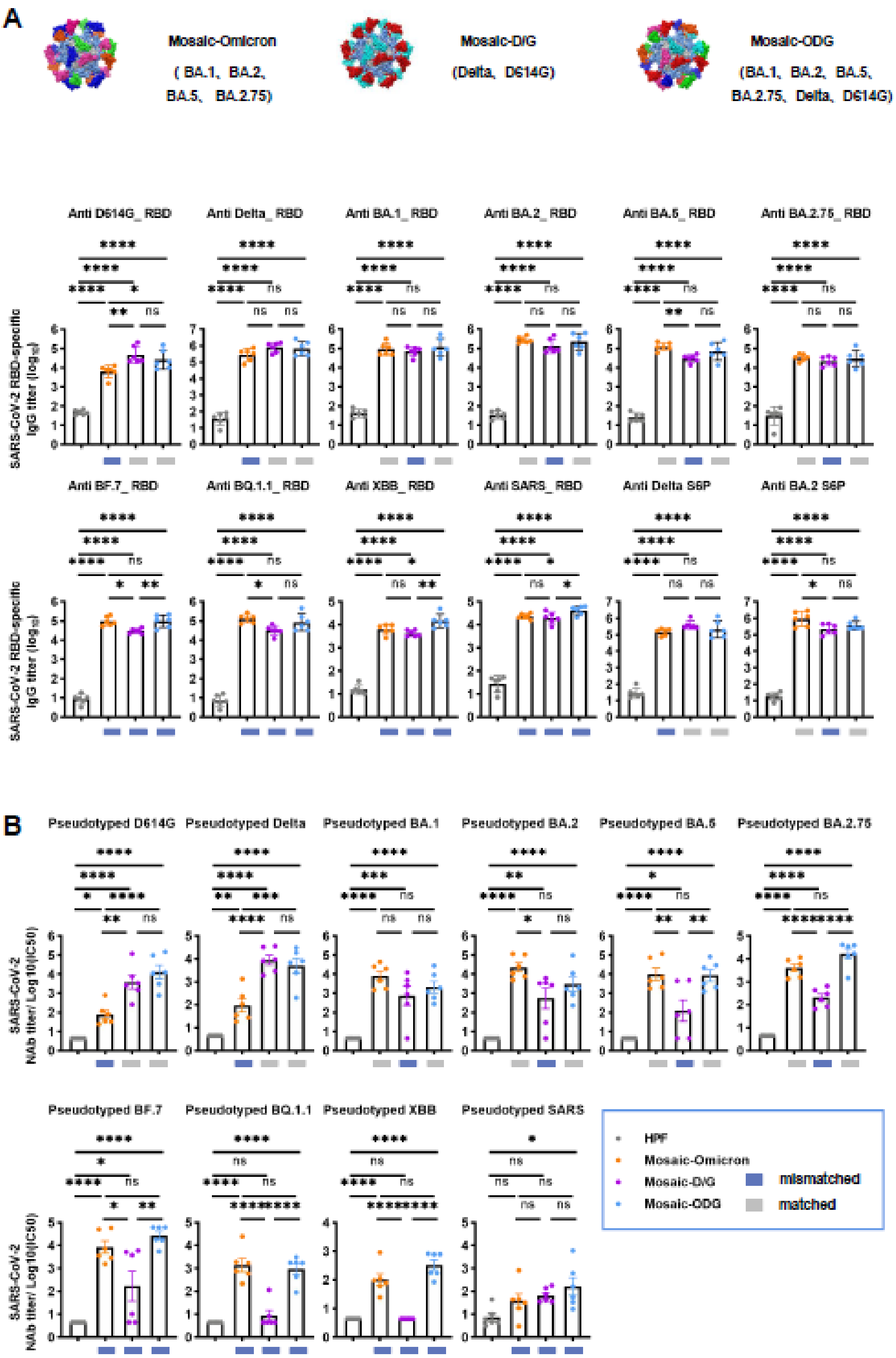

Figure S3. Mosaic-ODG-Np show broadly high-quality ELISA titers against the above sarbecovirus RBDs in BALB/c mice. BALB/c mice were immunized with

either 10μg Mosaic-Omicron, Mosaic-D/G, Mosaic-ODG, or the molar equivalent of HPF nanoparticles at week 0 and Week 4. **(A)** D614G RBD-, Delta RBD-, BA.1 RBD-, BA.2 RBD-, BA.5 RBD-, BA.2.75 RBD-, BF.7 RBD-, BQ.1.1 RBD-, XBB RBD-, SARS RBD-, Delta S6P-, and BA.2 S6P-specific IgG titers of immunized BALB/c mice at week six were detected by ELISA. IgG antibody titers of serum were determined by serial dilution, and the data are represented as the reciprocal of the endpoint serum dilution (n = 6). **(B)**Serialized diluted serum from vaccinated mice at week six was detected to neutralize antibodies against pseudotyped SARS-CoV-2 (D614G, Delta, BA.1, BA.2, BA.5, BA.2.75, BF.7, BQ.1.1, XBB） and SARS. The data represented NAbs NT50 within each group. Experiments were conducted independently in triplicates (n = 6). Rectangles below neutralization data indicate mismatched strains (blue; the RBD from that strain was not present on the nanoparticle) or matched strains (gray; the RBD was present on the nanoparticle). Data are represented as mean ± SEM. Adjusted p values were calculated by one-way ANOVA with Tukey's multiple comparisons test. Asterisks indicate significant differences between groups linked by horizontal lines. *p ≤ 0.05, **p ≤ 0.01, ***p ≤ 0.001, ****p ≤ 0.0001, ns = not significant.

fig. S4

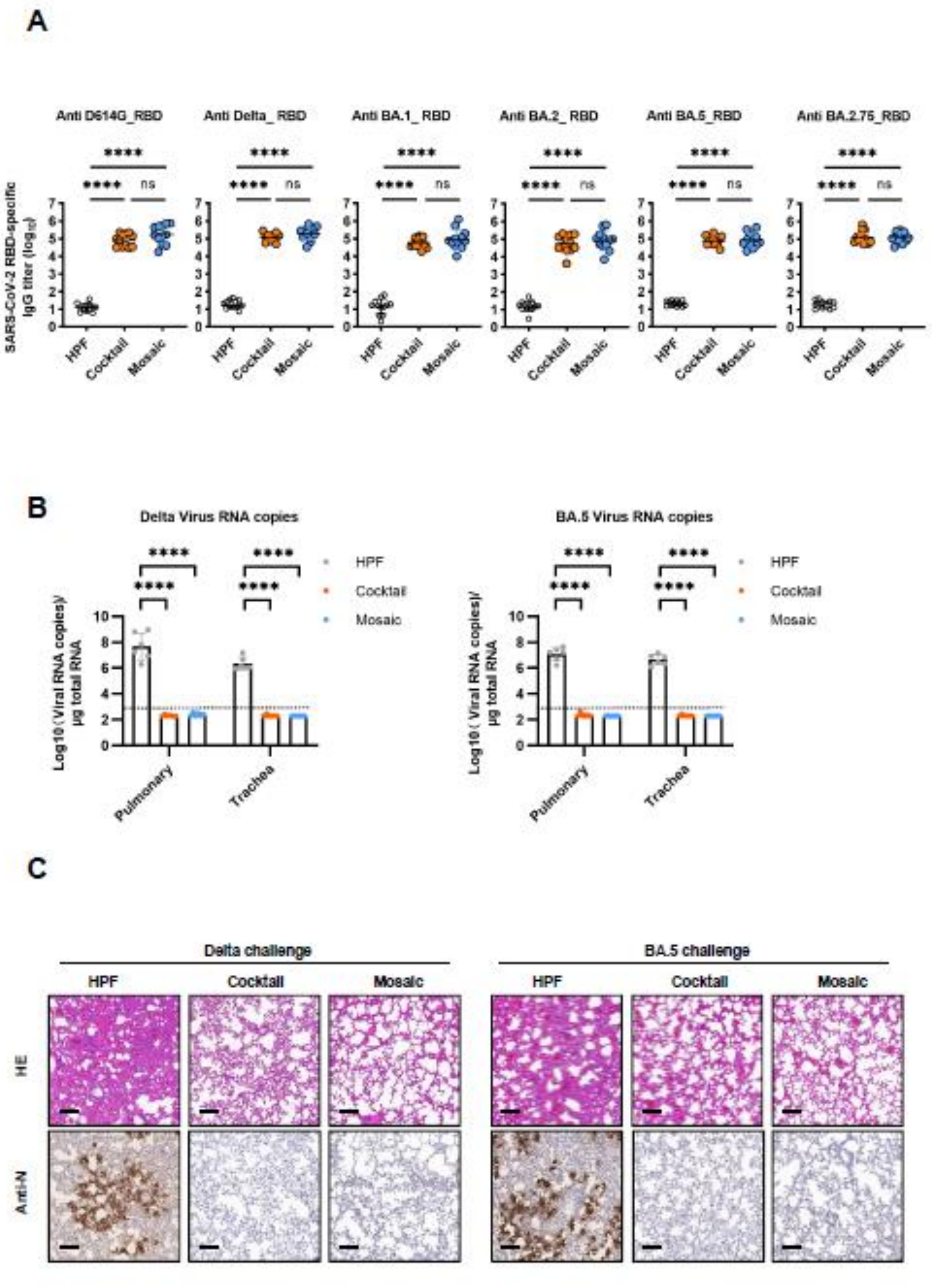

Figure S4. Mosaic RBD nanoparticles could induce strong humoral immune responses to protect against authentic variant infections in hACE2-K18 mice. (A)

D614G, Delta, BA.1, BA.2, BA.5, BA.2.75 RBD-specific IgG titers of immunized hACE2-K18 mice at week 6 were detected by ELISA (n = 12). **(B)** Viral RNA copies in the lungs and trachea of each mouse were determined by qRT-PCR and plotted as log10 copies per microgram. The dotted line indicates the limit of detection (LOD). Each dot represents a tissue sample from one animal (n = 6). **(C)** H&E staining and Immunohistochemistry (IHC) against N proteins was evaluated in the lungs of mouse. Scale bars represented 100 μm. Data are represented as mean ± SD. Adjusted p values were calculated by one-way ANOVA with Tukey's multiple comparisons test. Asterisks indicate significant differences between groups linked by horizontal lines. *p ≤ 0.05, **p ≤ 0.01, ***p ≤ 0.001, ****p ≤ 0.0001, ns = not significant.

fig. S5

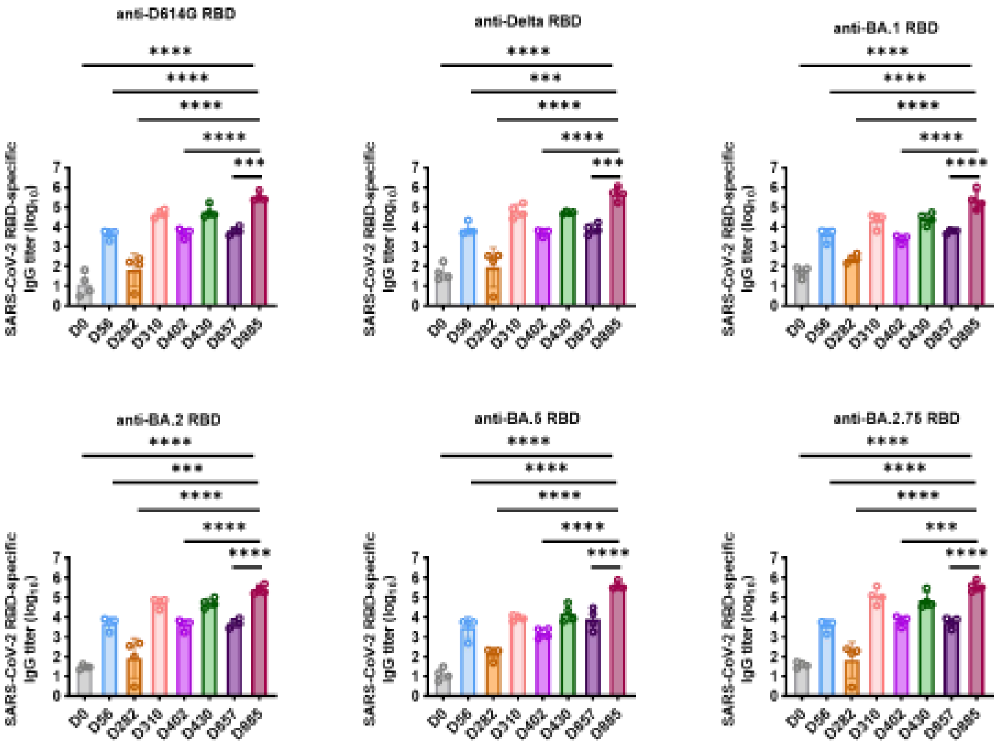

Figure S5. BA.1, BA.2, BA.5, BA.2.75, Delta, and D614G RBD-specific IgG titers of immunized rhesus macaque at different times were detected by ELISA. Data are represented as mean ± SD. Adjusted p values were calculated by one-way ANOVA with Tukey's multiple comparisons tests. Asterisks indicate significant differences

between groups linked by horizontal lines. *p ≤ 0.05, **p ≤ 0.01, ***p ≤ 0.001, ****p ≤ 0.0001, ns = not significant.

fig. S6

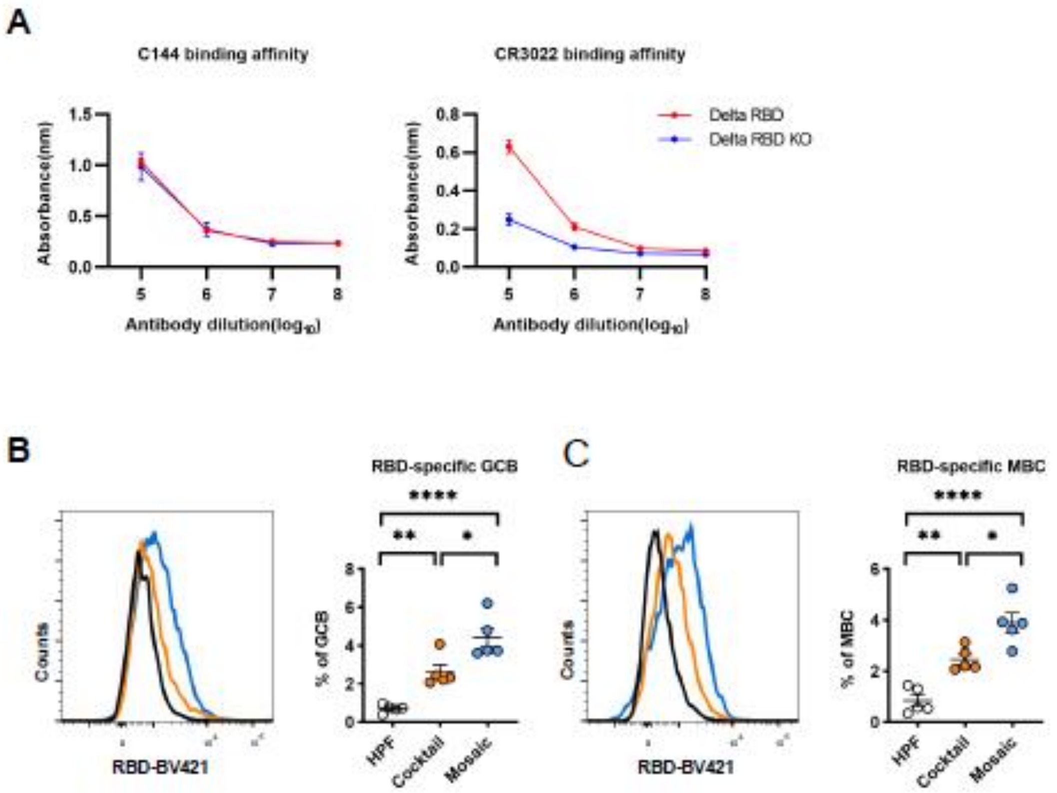

Figure S6. Mosaic vaccine elicits more RBD-specific humoral immune responses and Class IV antibody than a Cocktail vaccine in mice. (A) The binding affinity of Delta RBD-KO with C144 and CR3022 was evaluated by ELISA. **(B-C)** BALB/c mice were immunized with either 10 μg Mosaic RBD NP vaccine, Cocktail NP vaccine, or the molar equivalent of Sd-HPF NP through the subcutaneous route at Day 0 and Day 28, respectively. Two weeks post-immunization, mice were euthanized for the analysis of immune response. **(B)** The percentages of GC B cells ($CD19^+$ $CD95^+$ $GL7^+$) within the spleen of each vaccine group were determined by FCM. Antigen-specific GC B cells were further analyzed by a BV421-conjugated BA.2 RBD probe. **(C)** RBD-

specific MBC (CD19^{+} IgD^{-} CD38^{+} RBD-BV421^{+}) within the spleen of each vaccine group were determined by FCM. Data represented as mean ± SEM. P values were calculated by one-way ANOVA with Tukey's multiple comparisons test. *p ≤ 0.05, **p ≤ 0.01, ***p ≤ 0.001, ****p ≤ 0.0001, ns = not significant.

fig. S7

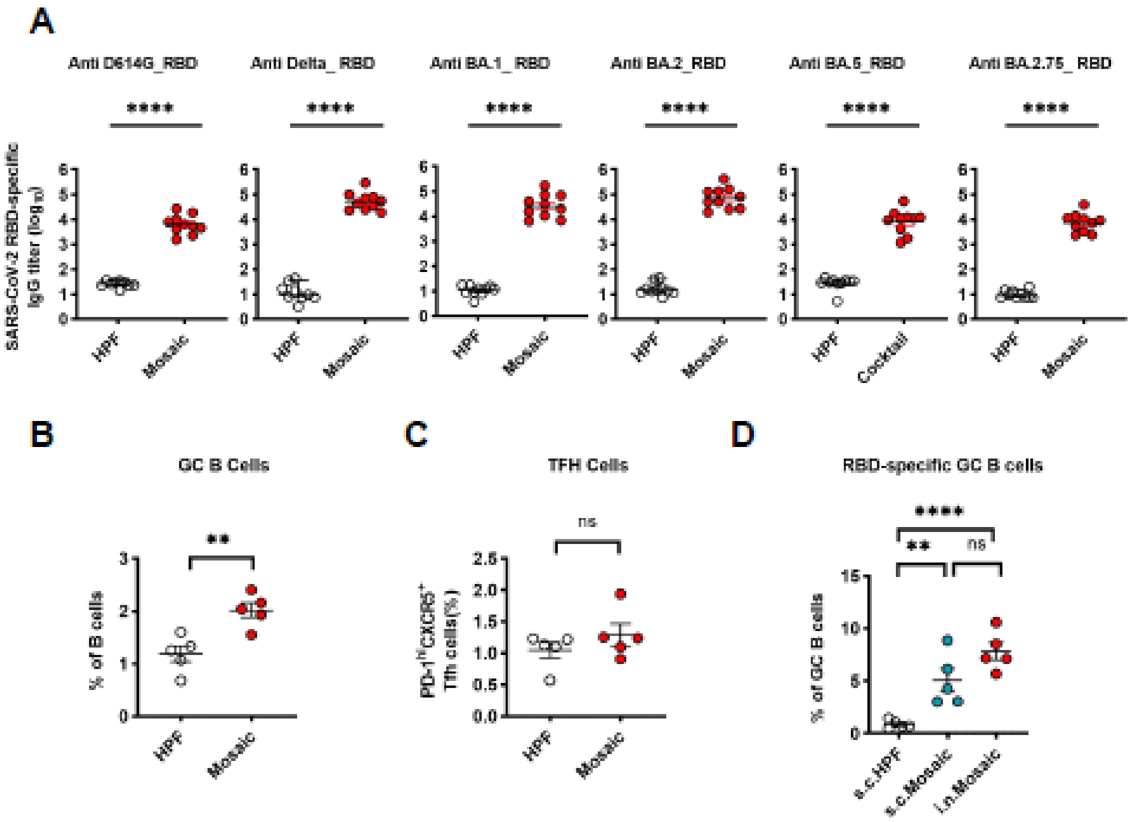

Figure S7. Intranasal vaccination with the Mosaic vaccine caused potent immune responses. (A) D614G, Delta, BA.1, BA.2, BA.5, BA.2.75 RBD-specific IgG titers of intranasally immunized hACE2-K18 mice at D35 were detected by ELISA. **(B-D)** Intranasal vaccination hACE2-K18 mice were euthanized for immune response analysis on day 35. The percentages of GC B cells **(B)** and TFH **(C)** within the spleen of each vaccine group were determined by FCM. **(D)** The percentages of RBD-specific germinal center B cells within the spleen in BALB/c mice, which were vaccinated with Mosaic RBD NP vaccine subcutaneously (s.c) or intranasally (i.n), were analyzed by flow cytometry. Data are represented as mean ± SEM. Adjusted p values were

calculated by one-way ANOVA with Tukey's multiple comparisons test. Asterisks indicate significant differences between groups linked by horizontal lines. *p ≤ 0.05, **p ≤ 0.01, ***p ≤ 0.001, ****p ≤ 0.0001, ns = not significant.

fig. S8

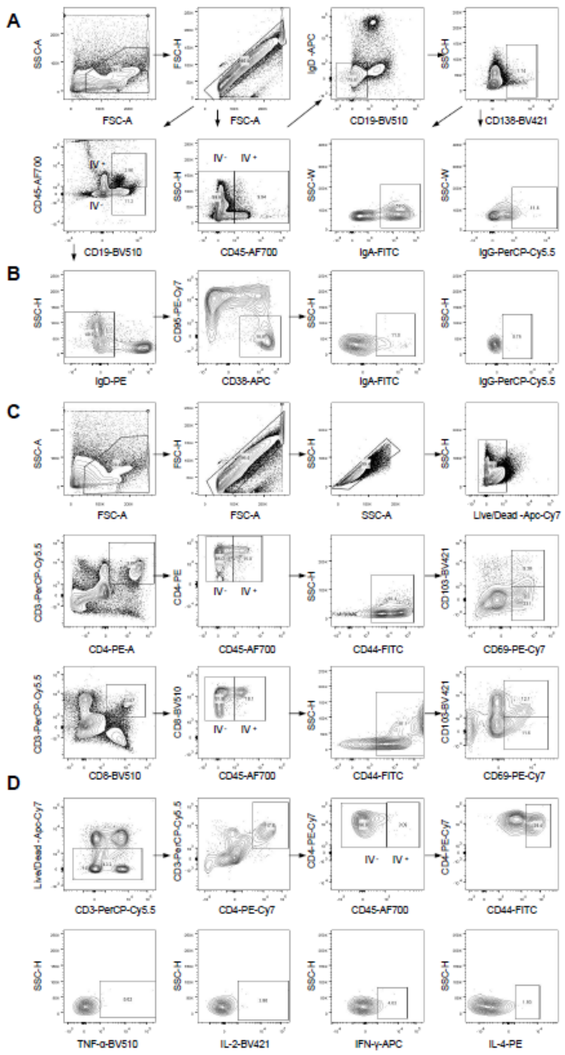

Figure S8. Gating strategies for analysis of adaptive immune responses in the respiratory tract.

(A) Gating strategies to identify plasma cells. **(B)** Gating strategies to identify B_{RM} cells. **(C)** Gating strategies to identify T_{RM} cells. **(D)** Gating strategy for intracellular cytokine staining in $CD4^+$ $CD44^+$ T cells.
